# Pruritus, Allergy and Autoimmunity: Paving the Way for an Integrated Understanding of Psychodermatological Diseases?

**DOI:** 10.3389/falgy.2021.688999

**Published:** 2021-09-17

**Authors:** Bárbara Roque Ferreira, José Luís Pio-Abreu, Américo Figueiredo, Laurent Misery

**Affiliations:** ^1^Laboratoire Interactions Epitheliums Neurones, University of Brest, Brest, France; ^2^Department of Dermatology, Centre Hospitalier de Mouscron, Mouscron, Belgium; ^3^Emeritus Professor of Psychiatry, Coimbra University, Coimbra, Portugal; ^4^Department of Dermatology, Coimbra University Hospital Centre, Coimbra, Portugal; ^5^University Hospital of Brest, Department of Dermatology, Brest, France

**Keywords:** allergy and immunology, autoimmunity, pruritus, psychological stress, skin diseases, psychodermatology

## Abstract

Pruritus is a key symptom in allergology and dermatology, contributing to the global and huge impact on quality of life related to skin disorders, both those which are not related to a primary dermatosis (illness) and those which are linked with primary skin lesions (disease). This is particularly evident within psychophysiological dermatoses, a group of psychodermatological diseases where there is a primary dermatosis, where psychological stress plays a role, and where pruritus may represent a major and shared symptom. The etiopathogenesis of pruritus in those disorders sheds light on the link among psychopathological features, psychological stress and the subtle interface between allergic and autoimmune mechanisms, where mast cells play a pivotal role. Allergy has long been recognised as an altered reactivity to exogenous antigens (allergens), defined as an immediate hypersensitivity mediated by immunoglobulin E (IgE). In turn, the immunological understanding of atopy is related to an immediate hypersensitivity reaction to environmental antigens involving T-helper 2 (Th2) responses and the IgE production. Mast cells are major cells in the early phase of allergy, releasing the mediators involved in the symptoms associated with the allergic disease, including pruritus, when the allergen cross-links with IgE, whose mechanisms can be observed in acute urticaria and atopy. Some allergic reactions may persist and allergy may eventually lead to autoimmunity, with the development of a T-helper 1 (Th1) and then IgE-independent inflammation. For instance, in chronic spontaneous urticaria, the mast cell activation may include autoimmune mechanisms, where autoantibodies against the extracellular α subunit of the high-affinity IgE receptor (FcεRIα) and to IgE are observed, with the involvement of Th1 lymphocytes and the production of interferon-γ (INF-γ). The role of autoimmunity is also suggested in the etiopathogenesis of other psychophysiological dermatoses, namely psoriasis, atopic dermatitis and alopecia areata. In the latter, for example, mast cells were reported to be linked with the loss of immune privilege and they are the key cells involved in the experience of pruritus, whose intensity was reported to precede and be correlated with the onset of the hair loss. Furthermore, considering that the role of hair and skin is wide, from psychosocial aspects (communication and social interaction) to vital functions (such as, temperature control), it is straightforward that they are central in our interactions and synchronization with others and the world; thereby, we may admit that the psychophysiological dermatoses could represent a loss of such synchronization. Furthermore, they are often linked with psychopathology which strongly connects with the concept of desynchronization, namely, sleep disorders and depressive symptoms, the clinical expression of a dysfunction in the interplay among mast cells, pineal gland and melatonin, thus the circadian rhythm, as well as their connection with the hypothalamic corticotrophin-releasing hormone (CRH), well-known for its key role in stress response. Moreover, increasing evidence has supported the existence of cutaneous equivalents for these mechanisms, connecting with those central pathways. Thereby, taking all these concepts into consideration, this review intends to look into the updated evidence on the shared biological mechanisms between allergy and autoimmunity, underlining pruritus as a core element, then revisiting the key role of mast cells and discussing the connection with melatonin and immune-inflammatory pathways in the physiopathology of psychophysiological dermatoses, thus paving the way for the understanding of their psychosomatic correlates and a comprehensive psychodermatological approach.

## Introduction

Pruritus, a common symptom in allergology and dermatology, belongs to the clinical expression of the excessive immune responses that may induce damage: when the immune system overreacts to innocuous environmental antigens, such as, in allergy, and to self-antigens, in the setting of autoimmunity. It is, thus, a core symptom in allergic and autoimmune dermatoses, suggesting that pruritus research may contribute to widen the understanding of the pathophysiological aspects linking allergy and autoimmunity (1, 2).

Furthermore, the study of pruritus is also relevant for a deeper understanding of the link between skin and psychological stress (3). In dermatology, there is a group of diseases where pruritus, autoimmune and immunoinflammatory mechanisms are shared and psychological stress plays a significant role both as trigger and for the clinical evolution. These dermatoses belong to the field of psychodermatology, where the disorders are broadly classified into four groups: primary psychopathology focused on the skin; psychophysiological dermatoses; cutaneous sensory disorders; skin diseases leading to secondary psychiatric illness (4). We herein discuss the group traditionally entitled “psychophysiological dermatoses,” which includes primary dermatoses that may be triggered or worsened by psychological stress, such as, alopecia areata, atopic dermatitis, chronic spontaneous urticaria and psoriasis (4). The main objective is to discuss clinical features shared by these psychodermatological diseases, considering their psychiatric comorbidities along with the physiopathology of pruritus, and the central role of mast cells, highlighting the slight boundary between allergy and autoimmunity.

## Psychodermatology and the Boundary Between Allergy and Autoimmunity

### Allergy and Autoimmunity: an Overview

Allergy, corresponding to an altered reactivity against exogenous antigens (allergens), is linked with immediate hypersensitivity reaction (type I) through immunoglobulin E (IgE), which needs T-helper 2 (Th2) cells that synthetize interleukin (IL)-4. Mast cells are the major cells involved in allergy, involved in the early phase through the release of mediators when IgE bound to the extracellular α subunit of the high-affinity IgE receptor (FcεRIα) is cross-linked by the allergen. Some allergic reactions may persist namely when the environmental antigen cannot be avoided and they can later lead to T cell infiltrate (5). Allergen sensitization has long been recognized as an early event in the etiopathogenesis of atopic dermatitis, involving a dysfunctional skin barrier, antigen presentation with subsequent mast cell and Th2 response (6). Although atopic dermatitis is associated with allergen-specific IgE, and other IgE-mediated allergic diseases, the role of autoreactive IgE is also recognized (7).

Autoimmunity represents an adaptive immune response whose specificity is targeted for self-antigens. Autoimmune disease may then start when autoreactive T cells or pathogenic antibodies, whose production also need T-helper 1 (Th1) response, induce tissue damage through hypersensitivity reactions II, III or IV. Additionally, human leukocyte antigen (HLA) alleles may play a significant role for the beginning of autoimmune mechanisms, regulating T cells (8). For example, a study conducted by Dogan et al. showed that HLA-DRB1 seems to be associated with chronic spontaneous urticaria, while HLA-A may prevent this dermatosis, which is in accordance with previous studies (9). Chronic spontaneous urticaria is linked with autoimmune mechanisms in at least 1/3 of cases, involving increased levels of CD4+ T cells and mast cells in both lesional and non-lesional skin, and seems to be highly positive for IL-17A (9, 10). Autoimmunity was firstly documented through in *vitro* studies, which showed the ability of the sera from patients with chronic spontaneous urticaria to provoke histamine release from basophils and cutaneous mast cells (11).

### Peripheral Tolerance and Autoimmunity: the Relevance in Psychodermatology

Another relevant topic concerning T cells and autoimmunity is tolerance: to induce an autoimmune disease, T cells need to surpass tolerance mechanisms. Apart from central tolerance, in the thymus, some autoreactive T cells might escape, but several peripheral mechanisms can still prevent these cells from responding. Among these mechanisms, there is the contribution of regulatory T cells (Tregs), which inhibit autoreactive T cells. Tregs can be specific for self-antigens as well as harmful environmental antigens (8).

Globally, Tregs are involved in both autoimmunity and allergy (12). Dysfunction of Tregs plays a central role in the etiopathogenesis of psychophysiological dermatoses, where autoimmune mechanisms are described, namely, chronic spontaneous urticaria, atopic dermatitis, psoriasis and alopecia areata (13–16). The relevance of the T cell immunoglobulin and ITIM domain (TIGIT) for Tregs response has been recently highlighted: an inhibitory receptor expressed on Tregs which suppresses T cell activation by the induction of regulatory dendritic cells. For instance, in atopic dermatitis, a reduction in TIGIT+ T cells may lead to a more severe clinical presentation (17) and the downregulation of this co-inhibitory receptor was also suggested in the pathogenesis of psoriasis and also related to the disease severity (18).

Another example of peripheral tolerance is the immunologic privilege that is observed, for instance, in the central nervous system (CNS) and in the hair follicle, where autoreactive T cells normally do not have access. When these mechanisms are compromised, autoimmunity may then develop. Anagen hair follicles present immunologic privilege from the bulge and downwards to the bulb. Along with Tregs, mast cells contribute to the hair follicle immunologic privilege (19). Mast cells, located in the perifollicular region, seem to play a functional dualism, both by having a pivotal role as a guardian of the immune privilege but also the ability to switch into a proinflammatory activity, influencing the activity of CD8+ T cells involved in the etiopathogenesis of alopecia areata (20). In alopecia areata, abnormal and increased physical contact between CD8+ T cells and mast cells is observed. Mast cells are also placed surrounding nerves in many tissues, including the skin, contributing to pruritus. Interestingly, pruritus seems to be a common reported symptom in patients with alopecia areata, prior to hair loss (21), and is correlated with mast cell activity (19). Thus, mast cells are central cells in autoimmune mechanisms as well as in IgE-mediated allergic diseases (22).

### Immune Responses in Psychodermatology: the Link Between Atopy and Autoimmunity

Globally, patients with atopy and those with other autoimmune diseases seem to have a higher likelihood to develop alopecia areata (21). Although interferon-γ (INF-γ)-secreting CD8+ T cells, promoted by CD4+ T cells, mast cells and dendritic cells, have a central role in the etiopathogenesis of alopecia areata (19), recent research has also pointed out the role of Th2 response, also explaining the association between alopecia areata and atopic dermatitis. In alopecia areata, Th1 response seems to be linked with disease onset and activity, while Th2 cytokines, with increased serum IgE and IL-4 levels, and increased levels of IL-17, were associated with long-standing disease (23).

Actually, in psychophysiological dermatoses, the immune responses are not usually so extreme and commonly involve a mix of Th1, Th2 and Th17 cells, in different proportions, also related to different clinical subgroups for the same dermatosis. For instance, in atopic dermatitis, Th2/Th22 is a shared signature across all ages, Th17 inflammation is increased in infants and Th1 polarization in adults (24). Patients with atopic dermatitis present a higher risk for both allergic and non-allergic diseases, including psychiatric disorders (25), and several autoimmune diseases, including alopecia areata, chronic urticaria, systemic lupus erythematosus and multiple sclerosis (7).

Furthermore, recent studies have documented that Th1, Th2 and Th17 cells, keratinocytes and mast cells may induce the production of IL-24, a pivotal cytokine in both allergic conditions and autoimmune diseases. Indeed, Il-24 can be induced by Th1-associated cytokines (INF-γ), Th2-associated cytokines (IL-4 and IL-31) and Th17 cytokine (IL-17A). Moreover, IL-24 can also be induced by classic pro-inflammatory cytokines with pleiotropic functions connecting innate and adaptive immunity, namely, IL-1, IL-6 and tumor necrosis factor-alpha (TNF-alpha), which can be also mast cell-derived (26) and have a key role in the activation of the hypothalamus-pituitary-adrenal (HPA) axis and its cutaneous equivalent, with release of corticotropin-releasing hormone (CRH), and then adrenocorticotropic hormone (ACTH) and corticosteroids (27). HPA axis is a well-recognized and key pathway through which psychological stress may lead to cutaneous inflammation (28), where IL-24 may play a central role, with relevance in the etiopathogenesis of pruritus and psychophysiological dermatoses. There is an upregulation of IL-24 expression in atopic dermatitis and in psoriatic epidermis, being a pivotal mediator in the development of both dermatoses and a key element in the immunological understanding of psoriasis-like features in atopic dermatitis skin. Furthermore, in chronic spontaneous urticaria, where IgE autoantibodies play an important etiopathogenic role, autoantibodies anti-IL24 IgE are frequent, correlate with pro-inflammatory cytokines and have a clinically significant association with disease severity. In turn, IL-24 can also induce mast cells to release histamine, thus acting as an autoantigen in this chronic dermatosis (29).

Thereby, the immunological subtleties shared by allergy and autoimmunity reinforce the interplay between innate and adaptive immune system components and the subtle connection among different dermatoses. Mast cells are a core element in both allergic (namely, atopy) and autoimmune mechanisms observed in psychophysiological skin diseases, where they also regulate the function of Tregs (30), whose dysfunction is a shared feature in the etiopathogenesis of those dermatoses (13–16). Altogether, the link between mast cell and T cell function is of central relevance in the understanding of cutaneous (neuro)inflammation, pruritus and psychodermatological disease (31).

## The Role of Mast Cells and Melatonin in Psychodermatology

### Mast Cell Mediators, Pruritus and Psychophysiological Dermatoses

Mast cells are pivotal cells in the understanding of pruritus in psychophysiological dermatoses and their neuropsychiatric comorbidities. They derive from a hematopoietic precursor cell triggered by Th2 cells-derived IL-3 and IL-4 and move to several tissues, including the skin, and they can also be resident cells, such as, in the skin and in the CNS. They produce a wide range of mediators, including, proteolytic enzymes, histamine, cytokines and arachidonic acid metabolites and participate in both innate and adaptive immune responses (32). Some mast cell mediators are produced upon activation, namely, important growth factors in neurogenic inflammation [such as, the nerve growth factor (NGF)] and cytokines with relevance in cutaneous inflammation (such as, IL-1, IL-6, IL-18, IL-31 TNF-alpha) (14, 32, 33). Among them, IL-31 has been highlighted as a key cytokine for pruritus in psychophysiological dermatoses, such as, psoriasis, chronic spontaneous urticaria, atopic dermatitis and alopecia areata (34).

Other mast cell mediators, with relevance in psychodermatology, are preformed: they are present in granules, from where they are released, such as serotonin (5-HT), histamine and tryptase. Mast cells may be activated via paracrine or autocrine pathways and move to critical regions in the CNS where they may influence neuronal activity, involving both neuroprotection and neurotoxic mechanisms and contributing to the physiopathology of psychiatric and neurodegenerative disease (32, 35).

5-HT, a biogenic amine, plays an immunoregulatory role in both CNS and peripheral nervous system (PNS), being involved in the sleep-wakefulness cycle, mood and behavior (32). Besides its role in the etiopathogenesis of common psychiatric comorbidities in psychodermatology, such as depression, 5-HT may also be relevant in the etiopathogenesis of pruritus in psoriasis and atopic dermatitis (36, 37). The link between serotonin and pruritus in those dermatoses may involve the serotonin-mediated activation of sensory neurons through cation channels, namely, TRPV4, through the 5-HT2 receptor, and TRPV1, through the 5-HT7 receptor (38–40).

Histamine, a major mediator in allergic diseases, classically associated with vascular permeability, tissue edema and pruritus, may have a much wider role, since this biogenic amine can have antagonistic neuronal effects, that is, neurotrophic, with neuroprotection, and neurotoxic effects. There is an interplay of histamine in neurons, glia, endothelial cells and mast cells, with an essential role in the regulation of several physiological mechanisms, including, circadian rhythms, mood and cognition. A dysfunction in that interplay seems to be involved in several neurologic and psychiatric diseases, where autoimmune mechanisms are recognized, such as Alzheimer's disease and schizophrenia (41). Nevertheless, although histamine and IgE remain important targets for the management of pruritus in the setting of chronic urticaria (42), its physiopathological role is less clear in atopic dermatitis and psoriasis (43), suggesting that it is not a major mediator for pruritus related to psychological stress.

In turn, proteolytic enzymes, such as, tryptase, induce inflammatory responses, activating the complement and kinin pathways, are involved in both allergic reactions and autoimmune mechanisms and may have an important role for the etiopathogenesis of pruritus linked with psychological stress. Tryptase is an endogenous proteinase-activated receptor-2 (PAR-2) agonist, which is increased in cutaneous nerve fibers in atopic dermatitis, contributing for cutaneous neurogenic inflammation and pruritus in this dermatosis (44). Its relevance was also well described in the physiopathology of pruritus related to psoriasis, alopecia areata and chronic urticaria (28, 45–47). For instance, in chronic spontaneous urticaria, tryptase levels are higher compared to controls and are greater in the autoimmune group compared to the idiopathic urticaria group and higher tryptase levels are associated with higher skin disease activity, as documented by several studies (46, 47). The increase in tryptase levels in patients with chronic spontaneous urticaria is also associated with a higher mast cell burden in the skin (47).

Although pruritus had remained relatively undervalued in psoriasis, increasing evidence has supported its relevance in this dermatosis, correlating with a significant impact on quality of life. Among other mechanisms, tryptase and kallikreins, which, as well as other cells, can also be derived from mast cells, are elevated in psoriatic skin, and seem to be involved in the pathways for pruritus, through the protease-activated receptor (PAR) family members (48). Psoriatic skin lesions also exhibit an increased mast cell count, and an elevated CRH expression, which may derive from cutaneous cells, including mast cells, keratinocytes and melanocytes (48).

### Mast Cells and Skin Stress Pathways

Skin residing mast cells also express CRH receptors (CRH-R1 and CRH-R2), thus having a paramount role in skin stress pathways, involved in cutaneous inflammation, as observed in psoriasis and other psychophysiological dermatoses. Mast cells that are located closed to nerve endings, whose density is enhanced in psoriatic skin, respond to psychological stress as a result of the increased levels of CRH. The pituitary adenylate cyclase-activating peptide (PACAP) is a stress mediator released from sensory and autonomic neurons and its receptors are also expressed on several cutaneous cells, including keratinocytes, mast cells and T cells. Enhanced levels of PACAP induce the activation of the HPA axis, with CRH production and then mast cell activity, triggering cutaneous neuroinflammation (48). In psoriatic skin, there is an increased expression of PACAP mRNA. Moreover, PACAP may also be involved in the etiopathogenesis of psychiatric comorbidities of psoriasis: depression and anxiety disorders. Additionally, PACAP-immunoreactivity was observed in cutaneous nerve fibers at the epidermis, the dermal-epidermal junction and surrounding blood vessels in psoriasis and close to dermal blood vessels and mast cells in patients with urticaria. In atopic dermatitis, immunoreactivity for PCAP receptors was observed in blood vessels, keratinocytes, mast cells and CD4+ T cells (49). Thus, PCAP may affect cutaneous vascular system, immune responses and mast cells (with the release of histamine and serotonin), T cells and keratinocytes activity, having thus a main role in neurovascular dysfunction and neurogenic inflammation (49), in several psychophysiological dermatoses.

Another classic stress mediator involved in the skin-nervous system interactions, is the calcitonin gene-related peptide (CGRP), expressed both in cutaneous sensory nerves and CNS. Higher CGRP serum levels were described in psoriasis and CGRP is also associated with psychiatric comorbidities, such as depression. Mast cells are an important target of stress mediators, including the neuropeptide CGRP, stimulating the release of vasoactive amines and T cell infiltrate (50). As in psoriasis, in atopic dermatitis, there is an increased density of CGRP- and substance P (SP)-positive nerve fibers, with higher epidermal and dermal mast cell-nerve fiber interactions and subsequent neurogenic inflammation. Although, in atopic dermatitis, CGRP plasma levels are not increased, compared to controls, patients with atopic dermatitis and intense pruritus have a significantly higher CGRP plasma level (50).

### Circadian Rhythm and Psychodermatology: A New Perspective and Future Directions

Several mediators are released upon psychological stress, such as CGRP, CRH, NGF and PACAP, and they act as mast cell activators, leading to the release or the synthesis of mast cell mediators, then triggering or worsening psychophysiological dermatoses (51). Therefore, in psychodermatology, mast cells are key cells in the etiopathogenesis of pruritus associated with psychological stress, being involved in the etiopathogenesis of the dermatoses and related psychopathology. Thereby, taking into account the physiological relevance of mast cells both in dermatology and neuropsychiatry, they might play a role in our synchronization with the others and the world and, thus, psychophysiological dermatoses, where depression and sleep disorders are characteristic comorbidities, could then represent a dysfunction of such synchronization, through a circadian rhythm disruption, as previously described for psychiatric illness. For instance, considering that the role of hair and the skin includes communication, social interaction, temperature control, psychophysiological dermatoses could be a result of such a desynchronization (52). This concept also brings about the reflection on the role of melatonin in psychodermatology, considering that it affects the activity of mast cells and T cells and plays a regulatory role in a wide range of physiological processes, related to the circadian rhythms, including the sleep-wake cycle, hormonal secretion and behavioral processes (53, 54). Furthermore, melatonin also has neuroprotective, anti-inflammatory and antioxidant effects and affects several skin functions, including the skin immune system, adnexal and barrier function, pigmentation, thermoregulation and regulation of vascular tone (53). Melatonin is derived from serotonine and its release is regulated by noradrenaline (53), two key neurotransmitters involved in depression. Depression and sleep disorders are models of loss of synchronization, related to circadian rhythm disturbances (54), and they are classic comorbidities of psychophysiological dermatoses (52). Although mainly produced in the pineal gland (“the third eye”), the human skin and the hair follicle also produce melatonin and express its receptors (53). In addition to the circadian rhythm control by the central regulator in the suprachiasmatic nucleus, which regulates melatonin secretion and is influenced by circulating melatonin (55), the skin also contains circadian clock genes, has endogenous rhythmicity and human hair follicle cycle, keratinocyte proliferation, cutaneous blood flow and transepidermal water loss exhibit circadian changes, regulated by melatonin, with relevance in several dermatoses, such as psoriasis and atopic dermatitis (56).

Thereby, melatonin may have a relevant role in autoimmunity and atopy (57), interfering with several T cell subsets, including, Tregs, Th1, Th2 and Th17 cells (58), thus being involved in the mechanisms behind the slight boundary between allergy and autoimmunity. As melatonin deficiency is correlated with sleep disorders (55) and depression severity (59), its imbalance may be involved in cutaneous inflammation and pruritus related to psychological stress, involving T-cell and mast cell dysfunction, a topic that deserves further research, with relevance in psychodermatology.

## Data Availability Statement

The original contributions presented in the study are included in the article/supplementary material, further inquiries can be directed to the corresponding authors.

## Author Contributions

BRF wrote the paper. JLP-A, AF, and LM reviewed it. All authors listed have made a substantial, direct and intellectual contribution to the work, and approved it for publication.

## Conflict of Interest

The authors declare that the research was conducted in the absence of any commercial or financial relationships that could be construed as a potential conflict of interest.

## Publisher's Note

All claims expressed in this article are solely those of the authors and do not necessarily represent those of their affiliated organizations, or those of the publisher, the editors and the reviewers. Any product that may be evaluated in this article, or claim that may be made by its manufacturer, is not guaranteed or endorsed by the publisher.
